# Draft Genome Sequences of Human-Pathogenic *Escherichia coli* O26:H11 Strains Carrying the *stx*_2_ Gene Only and Circulating in France

**DOI:** 10.1128/genomeA.00852-15

**Published:** 2015-07-30

**Authors:** Sabine Delannoy, Patricia Mariani-Kurkdjian, Stephane Bonacorsi, Sandrine Liguori, Sarah A. Ison, Patrick Fach

**Affiliations:** a Université Paris-Est, Anses, Food Safety Laboratory, Platform IdentyPath, Maisons-Alfort, France; b AP-HP, Hôpital Robert-Debré, Service de Microbiologie, CNR Associé *Escherichia coli*, Paris, France; c IAME, UMR 1137, INSERM, Paris, France; d IAME, UMR, Université Paris Diderot, Paris, France; e Department of Animal and Food Sciences, Texas Tech University, Lubbock, Texas, USA

## Abstract

Shiga toxin-producing *Escherichia coli* (STEC) O26:H11 is one of the most frequent pathogens associated with diarrhea and hemolytic-uremic syndrome (HUS). In this report, we present the draft genome sequences of seven strains of STEC O26:H11 carrying the *stx*_2a_ *or stx*_2d_ gene only and isolated in France from HUS patients.

## GENOME ANNOUNCEMENT

Among Shiga toxin-producing *Escherichia coli* (STEC) isolates, serotype O26:H11 is the second most frequently associated with severe human diseases worldwide, after O157:H7 (1, 2). Strains of STEC O26:H11 usually harbor the *stx*_1a_ gene only or in combination with *stx*_2a_. However, in the 1990s, a new clonal subgroup of STEC O26 emerged that carries the *stx*_2a_ gene alone (3). This new clone, first described in Germany, has spread over Europe and has recently been described on the American continent (4, 5).

In a previous study (6), we analyzed 23 STEC O26 strains carrying the *stx*_2_ gene only, which were isolated in France between 2010 and 2013 from hemolytic-uremic syndrome (HUS) patients. Although most of the strains appeared to correspond to this new clone, 12 of them exhibited significantly different characteristics.

In order to investigate more thoroughly the genetic relationship between the different clones, we sequenced seven of these strains covering the different combination observed between sequence types (ST), *stx* gene subtype, and virulence gene profile.

Genomic DNA was extracted from an overnight culture in tryptic soy broth (TSB) medium using the DNeasy blood and tissue kit (Qiagen), with an additional RNase A (Roche) treatment. Libraries were prepared using the Nextera XT kit (Illumina). Whole-genome sequencing was performed using an Illumina MiSeq platform (Illumina), according to the manufacturer’s instructions. Two MiSeq runs were carried out, one with paired-end 150-nucleotide (nt) reads on MiSeq V2 microchemistry and another with paired-end 300-nt reads on V3 chemistry. The raw reads were trimmed (minimum length, 35 bp; quality score, 0.03) and assembled in CLC Genomics Workbench 7.5.1 by *de novo* assembly (minimum contig length, 1,000 bp), producing 192 to 223 contigs (Table 1). The median read depth of the assemblies ranged from 38× for 34827 and 34870 to 84× for 36708, with an *N*_50_ between 65 kbp and 114 kbp (Table 1). The sequences were annotated using the National Center for Biotechnology Information (NCBI) Prokaryotic Genomes Automatic Annotation Pipeline (PGAAP) at http://www.ncbi.nlm.nih.gov/genomes/static/Pipeline.html.

**TABLE 1:** NCBI accession numbers and assembly metrics of the O26:H11 *E. coli* draft genomes

Isolate	ST	*stx* subtype	No. of contigs	Genome size (bp)	*N*_50_ (bp)	Median read depth (×)	No. of coding sequences (per PGAAP)	NCBI accession no.
36084	ST21	*stx*_2a_	205	5,235,007	96,218	45	5,209	LDXI00000000
36708	ST29	*stx*_2a_	192	5,526,827	114,644	84	5,579	LDXG00000000
34827	ST29	*stx*_2a_	223	5,604,044	73,150	38	5,674	LDXF00000000
34870	ST29	*stx*_2a_	196	5,498,010	65,024	38	5,524	LDXE00000000
36348	ST29	*stx*_2d_	208	5,570,467	94,008	71	5,573	LDXD00000000
36293	ST29	*stx*_2d_	207	5,458,923	75,621	45	5,460	LDXC00000000
36493	ST29	*stx*_2d_	204	5,422,929	78,448	47	5,389	LDXB00000000

The average size of the genomes in this study is 5.47 Mb, with 5.23 Mb being the smallest genome size (isolate 36084, Table 1) and 5.60 Mb the largest genome size (isolate 34827, Table 1). On average, 5,487 coding sequences were identified in the genomes (Table 1). BLAST analysis of the *stx* genes confirmed the previously identified subtypes (Table 1).

A detailed report on further analyses of the draft genome sequences will be released in a future publication.

### Nucleotide sequence accession numbers.

The annotated draft whole-genome sequences of these O26:H11 strains were deposited in DDBJ/ENA/GenBank under the accession numbers LDXB00000000 to LDXG00000000 and LDXI00000000 (Table 1). The versions described in this paper are the first versions, LDXB01000000 to LDXG01000000 and LDXI01000000.
